# Toxicity and Sublethal Effects of Diamide Insecticides on Key Non-Target Natural Predators, the Larvae of *Coccinella septempunctata* L. (Coleoptera: Coccinellidae)

**DOI:** 10.3390/toxics11030270

**Published:** 2023-03-16

**Authors:** Yunbo Cong, Jixiang Chen, Yinping Xie, Yingxiu Wang, Chunsheng Cheng

**Affiliations:** 1 School of Environmental and Chemical Engineering, Shenyang University of Technology, Shenyang 110870, China; 2 Key Laboratory for Chemical Pesticide of Shandong Province, Shandong Academy of Pesticide Sciences, Ji’nan 250100, China; 3 Shenyang Research Institute of Chemical Industry, Shenyang 110021, China

**Keywords:** *Coccinella septempunctata*, diamide, lethal effect, toxicity

## Abstract

*Coccinella septempunctata* (ladybird) is an extremely important natural predator that feeds on aphids. An assessment of the toxicity of pesticides on environmental organisms is an essential component of Integrated Pest Management (IPM) strategies. This study evaluated diamide insecticides’ toxicity at lethal and 30% lethal doses (LR_30_) against *C. septempunctata* larvae. The pre-imaginal median lethal doses (LR_50_) of chlorantraniliprole 10% SC, tetrachlorantraniliprole 10% SC, and broflanilide 10% SC were calculated to be 42.078, 289.516, and 0.0943 g active ingredient (a.i.)/ha, respectively. The mortality tests demonstrated that chlorantraniliprole and tetrachlorantraniliprole are comparatively less toxic to *C. septempunctata* than broflanilide, which were detected to be highly toxic to *C. septempunctata*. The mortality rates of the groups treated with the three diamide insecticides tended to stabilize after 96 h, extending to the pre-imaginal stage. Furthermore, when compared to broflanilide, which had a much higher potential risk, the hazard quotient (HQ) values indicated that chlorantraniliprole and tetrachlorantraniliprole have a lower risk potential for *C. septempunctata* in farmland and off farmland. The LR_30_ dose induces abnormalities in the development phase 4th-instar larvae weight, pupal weight, and adult weight of treated *C. septempunctata*. The study emphasizes the importance of assessing the adverse effects of diamide insecticides on natural predator species that serve as biological control agents in agricultural IPM strategies.

## 1. Introduction

From the past few decades to the present, agricultural pest control has relied heavily on applying chemical pesticides because of their practical and immediate response [1,2]. However, extensive and unmonitored usage of pesticides may cause adverse impacts on human health and the environment. Pesticides have been shown to have lethal and sublethal effects on beneficial arthropods [2,3,4]. The unique abundance of beneficial arthropod communities in the agroecosystem can be considered an important natural resource because it plays an important role in pest control. Further, the impact of insecticide application on these natural predators should be fully assessed to develop sustainably effective IPM programs.

The seven-spotted lady beetle, *Coccinella septempunctata* L. (Coleoptera: Coccinellidae), is a generalist predator in agroecosystems worldwide [5,6]. The larval and adult stages of *C. septempunctata* can effectively prey on aphid populations. Thus, *C. septempunctata* is an important natural enemy of pests with a voracious appetite and can rapidly respond to aphid populations in Integrated Pest Management programs (IPM) [7]. Furthermore, due to its high survival ability, rapid reproduction, and easy population expansion, *C. septempunctata* is used as an indicator test species to study integrated pest management and the adverse impacts of using natural predators in nature [8].

Diamide insecticides are a new class of pesticides with apparent advantages, like excellent pest control effects, long effective period, absence of cross-resistance with traditional pesticides, and ability to control various lepidopterans, hemipterans, and dipterous pests [9,10,11]. Regarding chemical structure, diamide insecticides can be divided into phthalic diamide, anthranilic diamide, and benzaminic diamide. Among these, flubendiamide, a phthalic diamide representative, was taken from the registration list because it poses a risk to aquatic life [9]. Anthranilic diamides have shown promising pest management effects. Anthranilic diamides were created to selectively activate insect ryanodine receptors. An uncontrolled release of calcium ions occurs when these receptors are activated, resulting in pest mortality [12]. Chlorantraniliprole was a commercial anthranilic diamide jointly developed by DuPont Pharmaceuticals (Wilmington, America). and Syngenta Co., Ltd. (Switzerland, Basilea) and has been proposed to be an alternative insecticide for the control of lepidopteran pests at lower application rates [13,14]. Tetrachlorantraniliprole is another novel ryanodine receptor modulator invented by Shenyang Chemical Research Institute Co., Ltd. (Shenyang, China) [2]. Its mechanism of action is similar to chlorantraniliprole, i.e., acting on the ryanodine receptors of muscle tissues and resulting in calcium release and death [15,16]. Broflanilide is a benzaminic diamide insecticide developed by Mitsui Chemical Agro, Inc. (Tokyo, Japan). and Badische Anilin- & Soda-Fabrik AG (Ludwigshafen, German) [17]. It was reported to have demonstrated an excellent effect on many pests. Broflanilides can block the inhibitory signals of insects, disrupt the nervous system, and causing continuous excitation, convulsions, and death by binding to the unique binding sites of the insect γ-aminobutyric and acid receptor (GABAR) [17,18,19]. Many studies have discovered that diamide insecticides are harmful to ladybird beetles and other non-target insects [20,21]. Therefore, the acute toxicity and sublethal effects of diamide insecticides on ladybird beetles have received increased attention. Understanding the impact of these insecticides on the biological characteristics of beneficial arthropods is crucial to improve the effectiveness of diamide insecticides in IPM systems. The present study used a standard dactylethrae contacting method to evaluate the acute toxicity and ecotoxicological risks of three representative diamide insecticides (chlorantraniliprole, tetrachlorantraniliprole, and broflanilide) on the larvae of *C. septempunctata* under laboratory conditions. Sublethal doses were also tested to assess their effects on a few developmental stages (developmental period of immature stages, pre-imaginal period, and pupae period) and body weight of *C. septempunctata*. The results would promote the conservation of ladybird beetles and provide available references for optimizing the use of diamide insecticides as a component of effective IPM strategies in agricultural ecosystems.

## 2. Materials and Methods

### 2.1. Chemicals

Chlorantraniliprole 10% SC, tetrachlorantraniliprole 10% SC, and broflanilide 10% SC were procured from the Key Laboratory for Chemical Pesticide of Shandong Province, China. Stock solutions were made by dissolving the substance in acetone before each experiment. From the stock solutions, various concentrations were obtained by dilution with acetone, and the test solutions has been provided in Table 1.

### 2.2. Test Species

Adults of *C. septempunctata* were collected from fields in the Shandong Academy of Agricultural Sciences, Jinan. They were reared under laboratory conditions without insecticide exposure for 8 to 10 generations before the experiment. The *C. septempunctata* were fed on aphids (*Aphis craccivora* Koch (Hemiptera: Aphidiae)) which were fed fresh broad bean seedlings under laboratory conditions, including a temperature of 25 ± 2 °C, 60 ± 10% relative humidity (RH), and 16:8 Light:Dark (L: D) photoperiods with an illumination intensity higher than 1000 lux. The test insects were 2nd-instar larvae with a consistent age of 12 h within the first ecdysis.

### 2.3. Bioassays

The methodology used for exposure was similar to that described by Jiang et al. [22]. Five test concentrations were chosen for each test compound, and 500 µL of the experimental solution was transferred into a glass tube (16.0 mm in diameter, 80.0 mm in height). The tubes were immediately rotated on a micro-rotator until the solvent had completely evaporated. Acetone was used in the case of blank controls. The design of the test has been provided in Table 1. The 2nd-instar larvae of *C. septempunctata* were individually introduced to the treated glass tubes within 12 h of molting and plugged with cotton. Ten larvae were exposed in a separate glass tube per replicate with three replications for each concentration. Sufficient aphids were provided daily according to the growth and age of the larvae. Every day, the number of deaths and other toxic symptoms were observed and recorded (death here is defined as an inability to crawl normally or an inability to react to mild touch).

### 2.4. Effects of LR_30_ on Larval Development and Weight

Based on the results of full dose–response bioassays, the effects of 30% sublethal doses (LR_30_) of diamide insecticides on the mortality, weight, and developmental duration of the 2nd-instar larvae of the *C. septempunctata* were further determined. The test procedures are similar to those described in Section 2.3, and mortality, weight, and developmental durations were recorded. The development data included the 2nd-, 3rd-, and 4th-instar periods, as well as pupal periods and pre-imaginal developmental time. The completion of ecdysis marked the end of larval development, and the pupal stage included the time between the completion of pupation and the completion of eclosion. The weight of insects at different observation points (6 h after the end of the third ecdysis, 12 h after pupation, and 12 h after eclosion) was recorded during the study.

### 2.5. Risk Assessment

The risk assessment of diamide insecticides against ladybirds was determined through the hazard quotient (HQ) method, which includes both the HQ in farmland (HQ_in_) and off farmland (HQ_off_) [23]. The HQ value is the ratio of the predicted exposure concentration (PEC) in the field to the median lethal doses (LR_50_) determined in the laboratory. The HQs of less than or equal to 5 were considered acceptable, while higher ratios indicated a potential risk to *C. septempunctata*.

### 2.6. Statistical Analysis

The LR_50_ was determined by probit analysis using SPSS software. The significance of the differences between the different insecticides was determined according to the cephalocaudal overlapping of 95% confidence intervals (version 25.0, SPSS Inc., Chicago, IL, USA) [24]. The total developmental duration and survival probability were compared across instars using one-way ANOVA followed by Tukey’s honest significant difference (HSD) test (*p* < 0.05).

## 3. Results

### 3.1. Acute Toxicity of Diamide Insecticides in C. septempunctata

On exposure to the insecticides, the larvae began showing rigidity and incoherent movements until the treated insects died of dehydration. Dehydration killed the majority of insects during the pupal period. The feeding and activity of the ladybirds were normal, and the mortality rate in the control group was less than 10%.

**Figure 1:**
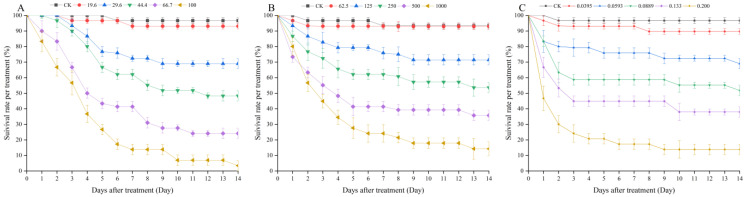
Effects of three diamide insecticides on the survival rate of *C. septempunctata* larvae during the 14 d observation period before pupation (**A**) chlorantraniliprole 10% SC; (**B**) tetrachlorantraniliprole 10% SC; (**C**) broflanilide 10% SC). Data are represented as mean ± SE (ANOVA, Tukey’s LSD, *p* < 0.05).

The LR_50_ values were calculated at 48 h, 96 h, prepupa, and pre-imaginal larvae periods of *C. septempunctata* after exposure to the three classes of diamide insecticides and have been provided in Table 2. The pre-imaginal LR_50_ of chlorantraniliprole 10% SC, tetrachlorantraniliprole 10% SC, and broflanilide 10% SC were recorded to be 42.078, 289.516, and 0.0943 g active ingredient (a.i.)/ha, respectively. The toxicity of broflanilide was significantly higher than that of chlorantraniliprole and tetrachlorantraniliprole to *C. septempunctata*. In addition, the LR_50_ values of the three diamide insecticides were affected due to the duration of exposure. With the increase in exposure time, the survival rate gradually decreased with a gradual rise in toxicity (Figure 1). For chlorantraniliprole 10% SC and tetrachlorantraniliprole 10% SC, the mortality rates were recorded to be lower on days 1 and 2. Even at the highest concentrations, the mortality rate of treated larvae was less than 50%. The mortality rates at days 3 and 4 increased dramatically, while the rates at days 5 and 6 increased only slightly. The survival rates stabilized on day 7 after exposure. There were no significant differences among the LR_50_ of 48 h, 96 h, prepupa, and pre-imaginal periods. The mortality of *C. septempunctata* treated with broflanilide 10% SC at the three highest concentrations started to decline rapidly after day 1. By day 3 post-exposure, the mortality rates were stable. During exposure, the survival rate declined but gradually stabilized after the prepupal stage. A few pupae failed to emerge from the developmental stage, and an unpupated larva was regarded as a larval death.

The risk assessment was carried out using the LR_50_ in the pre-imaginal stage and is reported in Table 3. In the simulated field application scenario, the HQ_in_ values of chlorantraniliprole 10% SC and tetrachlorantraniliprole 10% SC for the individual larva were recorded to be 1.43 and 0.207, respectively, and the HQ_off_ for the individual larva were recorded as 0.0339 and 0.00574, respectively. All values were less than the risk ratio threshold of 5. Therefore, practical usage guidelines could establish that ladybird beetles face no risk of insecticide exposure. After applying broflanilide 10% SC to the field, the HQs for the individual larva were 254 in farmland and 7.05 off farmland, exceeding the threshold risk ratio of 5. These data indicate a potential toxicity risk from broflanilide exposure.

### 3.2. Sublethal Effects on the Larval Developmental Period

The sublethal effects of the three insecticides on the development period of different life stages of *C. septempunctata* are reported in Table 4. These results show that the duration of development stages as well as the overall duration of treatments were slightly longer compared to the control group (F3, 8 = 16.97, *p* = 0.001; F3, 8 = 5.87, *p* = 0.020; F3, 8 = 5.22, *p* = 0.027; F3, 8 = 17.58, *p* = 0.010, F3, 8 = 41.81, *p* < 0.001 for 2nd-, 3rd-, and 4th-instar larvae, pupae, and total developmental time, respectively). When broflanilide 10% SC was used instead of chlorantraniliprole 10% SC or tetrachlorantraniliprole 10% SC, the 2nd-instar larvae period and the entire developmental period were significantly longer. Although the same trend in the duration of development was observed for 3rd- and 4th-instar larvae, the difference was not significant. Moreover, among all treatments, only broflanilide 10% SC treatments significantly increased the pupal duration. These results revealed that the three diamide insecticides significantly negatively affected the development of the 2nd-instar *C. septempunctata* larvae.

**Figure 2:**
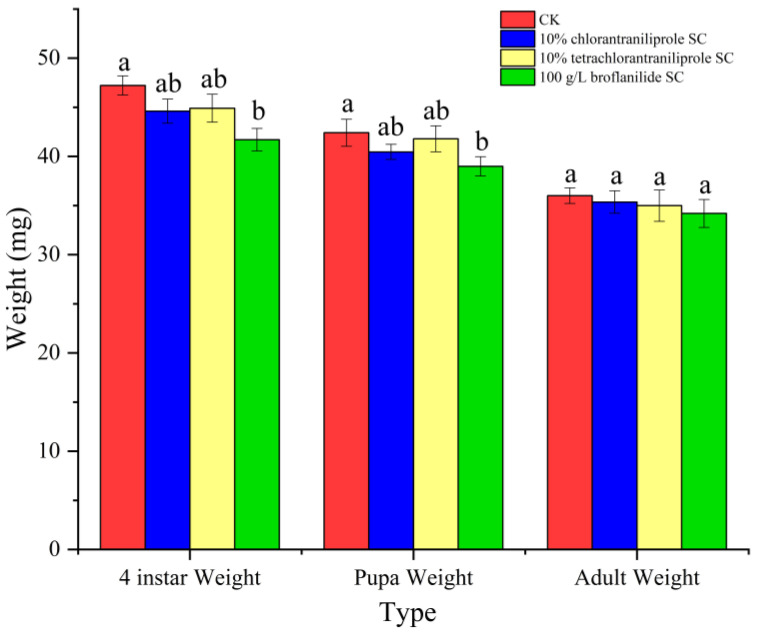
The weight of the larvae, pupae, and adults treated with three diamide insecticides (mean value relative to ‘individual’ weight in each test system and error bars). Data from the same period are marked with different lowercase letters and are significantly different (ANOVA, HSD test, *p* < 0.05).

The weights of the larvae, pupae, and adults treated with the three diamide insecticides were lower than those of the control group. After treatment with broflanilide 10% SC, 4th-instar larvae weight and pupal weight were considerably lower than in the control group, but there were no significant differences after treatment with chlorantraniliprole 10% SC and tetrachlorantraniliprole 10% SC (*p* = 0.004, 0.028, respectively). After the three pesticide treatments, adult weights were lower than in the control group, although there were no statistically significant differences (*p* > 0.05) (Figure 2).

## 4. Discussion

Because of their new targets, effectiveness, and broad range, diamide insecticides are essential for reducing residual risk, decreasing pest resistance, and increasing agricultural productivity [11]. However, there are some drawbacks to these highly bioactive pesticides. They pose significant dangers to ecosystems because of their high toxicity to specific non-target organisms, long persistence in soil, water, etc. [25]. The dactylethrae contacting method was used in our study to assess the acute toxicity of three representative diamide insecticides on *C. septempunctata* larvae. The three insecticides used in the present study exhibited different modes of action. Therefore, differences between the two types of products were observed in their activity on the same predator species. Chlorantraniliprole and tetrachlorantraniliprole did not exhibit acute toxicity against the larvae of *C. septempunctata*, because its LR_50_ for larvae (42.078 g a.i./ha and 289.516 g a.i./ha) were higher than the recommended application doses (40 g a.i./ha and 60 g a.i./ha) in the field. Similarly, chlorantraniliprole has been proven safe for *C. septempunctata* [13], and tetrachlorantraniliprole caused no lethal effects for *Harmonia axyridis* [16]. Compared with chlorantraniliprole and tetrachlorantraniliprole, the broflanilide treatment had a different lethal effect on *C. septempunctata*. It has been recorded that broflanilide acts quickly in larval *C. septempunctata* after 24 h, and the LR_50_ for larvae (0.0943 g a.i./ha) was significantly lower than the recommended field rates (24 g a.i./ha) in the field. The mortality of larvae of *C. septempunctata* increased with broflanilide concentrations and exhibited a dose–response relationship.

Insecticides are unevenly distributed and gradually degraded when applied, increasing the likelihood of insects coming into contact with low concentrations of insecticides [22]. Therefore, research on the sublethal effects of insecticides on insects can improve pesticide rationalization and protect non-target insects [24]. Previous studies showed that sublethal doses of chlorantraniliprole reduced the larval development time, adult weight, longevity, fecundity, and daily predation in *C. septempunctata* [13], and delayed the reproductive maturation and reduced the fecundity in *Daphnia magna* [9]. However, Teng et al. reported that tetrachlorantraniliprole did not affect *H. axyridis ladybugs* and *E. fetida* earthworms [16]. There is little information available on the environmental behavior and risk assessment of broflanilide. Broflanilide acute toxicity in *Danio rerio* is low; however, it is worth noting that broflanilide could rapidly accumulate in *D. rerio* in sub-lethal concentrations after a short time of exposure [20]. Our toxicity findings showed that *C. septempunctata* development and larval stages were delayed by sublethal doses of the three diamide insecticides. The larvae and pupae developmental results presented here show that tetrachlorantraniliprole is toxic even at low concentrations during pre-adult development. The development time for pre-adults was significantly increased in diamide-treated insects compared to the control group, probably because insecticide-treated larvae need to devote more of their energy to chemical detoxification [26]. Additionally, we found that the three diamide insecticides reduced the larval weight, pupal weight, and adult weight of *C. septempunctata*. These findings support previous research on the effects of diamide insecticides on other natural enemies [13,16]. Long-term toxic effects on aspects such as growth and development, reproduction, and offspring may occur in addition to acute toxicity. These effects could result in pesticide resistance, fewer offspring, decreased reduced effectiveness, and lower population levels of these natural enemies in agricultural ecosystems [27].

The risks of chemicals on environmental organisms (including predators, parasitoids, and aquatic algae) can be assessed by several environmentally relevant endpoints [28], which can be used to prove the margin of safety for pesticides on environment [27,29]. The LR_50_ at different observation points was significantly different. For chlorantraniliprole 10% SC, the survival rate tended to stabilize after the prepupae stage. The LR_50_ at the pre-imaginal stage was significantly different compared to the larval stage (48 h and 96 h after exposure). Thus, using the pre-imaginal stage as an endpoint for toxicity determination was more accurate than previous studies using specific time points such as 24 h or 72 h. Our HQ values indicated that chlorantraniliprole and tetrachlorantraniliprole (HQ < 5) are relatively safe for *C. septempunctata* at the recommended application doses. Conversely, broflanilide (HQ > 5) can be strongly toxic to predators by contact experiments. These results revealed that diamide insecticides had selective toxicity to *C. septempunctata*, which means that diamide insecticide applications need to be assessed on a case-by-case basis.

The results showed that there were differences in the sensitivity of *C. septempunctata* larvae to the three diamide pesticides. Despite their chemical relatedness, their toxicity to *C. septempunctata* varied. An insecticide’s toxicity level in a bioassay depends on the chemical group and the test target insect species [30]. Therefore, the toxicity difference between broflanilide and the other two insecticides (chlorobenzamide and tetrachlorofenamide) to *C. septempunctata* may be caused by their chemical group differences. In addition, aside from the toxicity of direct exposure, the consumption of insecticide-treated aphids during the experiment was also an essential factor in the toxicity levels of pesticide types and susceptibilities of *C. septempunctata* [31].

## 5. Conclusions

This study evaluated the acute toxicity of three diamide insecticides to *C. septempunctata* by the dactylethrae contacting method. The results showed that the toxicity levels of different diamide pesticides against *C. septempunctata* larvae differ significantly. The toxicity of benzaminic diamides (broflanilide) was significantly higher than that of anthranilic diamides (chlorantraniliprole and tetrachlorantraniliprole). Various diamides influenced developmental duration after treatment with diamide insecticides at sublethal doses. After treatment with the three diamide insecticides, the ladybird beetle’s predation ability was lower than that of the control group based on body weight and developmental duration at different developmental stages. In our study, broflanilide was found to be a potential risk to ladybird beetles. Therefore, the adverse impacts of broflanilide on *C. septempunctata* should be considered when broflanilide is used to control agricultural pests. At the same time, when releasing natural predators such as ladybird beetles for biological control, the use of broflanilide should be reduced.

## Figures and Tables

**Table 1:** The test solutions of three diamide insecticides.

Treatment	Chlorantraniliprole 10% SC	Tetrachlorantraniliprole 10% SC	Broflanilide 10% SC
Dosage (μg/mL)	23.3	74.1	0.0468
	35.0	148	0.0702
	52.4	296	0.105
	78.7	592	0.158
	118	1185	0.237

**Table 2:** The half-lethal dose (LR_50_) of three diamide insecticides in the larvae of *C. septempunctata*.

Insecticide	Exposure Time	Toxic Regression Equation	LR_50_/(g a.i./ha)	95% Confidence Interval	R^2^
chlorantraniliprole 10% SC	48 h	-	-	-	-
	96 h	Y = 3.058X − 5.717	74.037	66.654–84.227	0.979
	Prepupa	Y = 3.477X − 5.831	47.536	43.744–51.746	0.991
	Pre-imaginal	Y = 4.186X − 6.799	42.078	39.097–45.247	0.985
tetrachlorantraniliprole 10% SC	48 h	-	-	-	-
	96 h	Y = 1.492X − 4.016	491.969	404.355–624.628	0.986
	Prepupa	Y = 1.774X − 4.511	349.428	298.117–415.074	0.989
	Pre-imaginal	Y = 1.931X − 4.754	289.516	249.790–337.076	0.984
broflanilide 10% SC	48 h	Y = 2.868X + 2.539	0.130	0.118–0.147	0.954
	96 h	Y = 3.097X + 2.948	0.112	0.102–0.124	0.991
	Prepupa	Y = 2.951X + 2.872	0.106	0.0967–0.118	0.984
	Pre-imaginal	Y = 3.051X + 3.128	0.0943	0.0860–0.104	0.982

**Table 3:** Calculation of HQs of three diamide insecticides of *C. septempunctata*.

Pesticides	Pre-imaginal LR_50_/ (g a.i./ha)	AR/ (g a.i./ha)	*n*	*i*	MAF	VDF	PDF%	HQ_in_	HQ_off_
chlorantraniliprole 10% SC	42.078	40	2	10	1.50	5	2.38	1.43	0.0339
tetrachlorantraniliprole 10% SC	289.516	60	1	/	1.00	5	2.77	0.207	0.00574
broflanilide 10% SC	0.0943	24	1	/	100	5	2.77	254	7.05

**Table 4:** The effect of sublethal dose (LR_30_) of three diamide insecticides on the developmental duration of *C. septempunctata*.

Treatment	2nd-Instar Developmental Time (Days ± SE)	3rd-Instar Developmental Time (Days ± SE)	4th-Instar Developmental Time (Days ± SE)	Pupae Developmental Time (Days ± SE)	Total
CK	2.43 ± 0.0557 c	2.68 ± 0.0872 c	4.60 ± 0.1069 c	4.79 ± 0.0529 b	14.50 ± 0.0451 c
chlorantraniliprole 10% SC	2.65 ± 0.0710 b	3.03 ± 0.1665 ab	4.68 ± 0.0710 b	5.19 ± 0.0416 a	15.55 ± 0.1249 b
tetrachlorantraniliprole 10% SC	2.67 ± 0.0586 b	2.93 ± 0.1795 ab	4.68 ± 0.1301 ab	5.26 ± 0.1168 a	15.54 ± 0.3421 b
broflanilide 10% SC	2.86 ± 0.1015 a	3.13 ± 0.0961 a	4.91 ± 0.0751 b	5.39 ± 0.1652 a	16.29 ± 0.1418 a
F	16.97	5.87	5.22	17.58	41.81
Df	3, 8	3, 8	3, 8	3, 8	3, 8
*p*	0.001	0.020	0.027	0.010	<0.001

Data are presented as the mean value ± SD (standard deviation), and means followed by the same letters in the same column are not significantly different (one-way ANOVA and Tukey test, *p* < 0.05).

## Data Availability

All data are available upon request from the authors.
